# Nanopore sequencing of clonal IGH rearrangements in cell-free DNA as a biomarker for acute lymphoblastic leukemia

**DOI:** 10.3389/fonc.2022.958673

**Published:** 2022-12-14

**Authors:** Shilpa Sampathi, Yelena Chernyavskaya, Meghan G. Haney, L. Henry Moore, Isabel A. Snyder, Anna H. Cox, Brittany L. Fuller, Tamara J. Taylor, Donglin Yan, Tom C. Badgett, Jessica S. Blackburn

**Affiliations:** ^1^ Department of Molecular and Cellular Biochemistry, University of Kentucky, Lexington, KY, United States; ^2^ Markey Cancer Center, University of Kentucky, Lexington, KY, United States; ^3^ College of Medicine, University of Kentucky, Lexington, KY, United States; ^4^ Department of Pediatric Oncology, University of Kentucky, Lexington, KY, United States; ^5^ Department of Biostatistics, University of Kentucky, Lexington, KY, United States

**Keywords:** B-cell acute lymphoblastic leukemia (B-ALL), central nervous system (CNS) disease, clonality, minimal residual disease (MRD), relapse, VDJ rearrangement

## Abstract

**Background:**

Acute Lymphoblastic Leukemia (ALL) is the most common pediatric cancer, and patients with relapsed ALL have a poor prognosis. Detection of ALL blasts remaining at the end of treatment, or minimal residual disease (MRD), and spread of ALL into the central nervous system (CNS) have prognostic importance in ALL. Current methods to detect MRD and CNS disease in ALL rely on the presence of ALL blasts in patient samples. Cell-free DNA, or small fragments of DNA released by cancer cells into patient biofluids, has emerged as a robust and sensitive biomarker to assess cancer burden, although cfDNA analysis has not previously been applied to ALL.

**Methods:**

We present a simple and rapid workflow based on NanoporeMinION sequencing of PCR amplified B cell-specific rearrangement of the (IGH) locus in cfDNA from B-ALL patient samples. A cohort of 5 pediatric B-ALL patient samples was chosen for the study based on the MRD and CNS disease status.

**Results:**

Quantitation of IGH-variable sequences in cfDNA allowed us to detect clonal heterogeneity and track the response of individual B-ALL clones throughout treatment. cfDNA was detected in patient biofluids with clinical diagnoses of MRD and CNS disease, and leukemic clones could be detected even when diagnostic cell-count thresholds for MRD were not met. These data suggest that cfDNA assays may be useful in detecting the presence of ALL in the patient, even when blasts are not physically present in the biofluid sample.

**Conclusions:**

The Nanopore IGH detection workflow to monitor cell-free DNA is a simple, rapid, and inexpensive assay that may ultimately serve as a valuable complement to traditional clinical diagnostic approaches for ALL.

## Introduction

Acute Lymphoblastic Leukemia (ALL) is the most common pediatric malignancy. Despite cure rates approaching 90%, relapsed ALL remains the second leading cause of cancer-related death in the pediatric population. Clinicians measure response to chemotherapy after the first round of treatment by assessing the number of leukemic blasts remaining after the first round of treatment. The presence or absence of minimal residual disease (MRD), or more than 0.01% of leukemic blasts remaining at the end of induction and consolidation chemotherapy, remains the most powerful prognostic indicator of patient outcome (1–4). The central nervous system (CNS) is also an important site of involvement in ALL and is a common site for ALL relapse (5, 6). The prevalence of CNS disease in ALL is high enough that every ALL patient receives prophylactic treatment involving multiple doses of intrathecal chemotherapy, which can have adverse and long-term side effects in pediatric patients. Better stratification of patients based on risk status and potential for relapse or CNS disease will rely on more precise diagnostics.

Current methods to diagnose MRD and CNS disease in ALL rely on detecting blasts in patient samples using flow cytometry or microscopy. Developing more sensitive assays that depend on the molecular detection of leukemic blasts *via* next-generation sequencing (NGS) has proven difficult since ALL has one of the lowest mutational burdens of any cancer (7, 8). This lack of common mutations precludes the design of targeted, cancer-specific sequencing probes commonly used in NGS assays for other types of cancers. Recently, Adaptive Biotechnologies developed the ClonoSEQ assay, an NGS assay that sequences VDJ rearrangements within B cell receptors instead of cancer-specific mutations (9, 10). This strategy is ideal for lymphocytic leukemias, as every T and B cell undergoes VDJ recombination to generate functional B and T cell receptors; the specific sequence of the rearrangement in the receptor is unique to an individual B or T cell. ALL results from the abnormal expansion of typically 1-5 B or T cell clones, each of which will have a unique VDJ sequence (11, 12). ClonoSEQ uses NGS methods to sequence the genomic DNA of cells isolated from patient blood and bone marrow samples to identify clonally expanded VDJ sequences and detect as few as one leukemic blast per million cells. ClonoSEQ has been implemented in Europe for MRD detection in B-cell lymphoid malignancies and is FDA-approved and in clinical trials in the US (13–15).

The major limitation of the ClonoSEQ method is its reliance on the collection of genomic DNA from leukemia cells within the patient sample. A significant concern for any cell-based assay is the accurate detection of CNS disease in ALL. Animal models showed that ALL cells often become embedded in the CNS tissue or attached to spinal nerves (16, 17). Approximately 85% of patients that succumbed to their disease had ALL blasts present in the brain or spinal cord at autopsy (18). However, only 11% of patients are clinically diagnosed as CNS disease positive, based on the detection of lymphoblasts in their cerebrospinal fluid (CSF) (5, 6). These findings highlight a critical need to develop more sensitive assays to complement cell-based pathology, flow cytometry, and ClonoSEQ assays, especially pertaining to ALL spread into the CNS.

Cell-free DNA as a cancer biomarker is transforming how patients are diagnosed and monitored for cancer progression. Cell-free DNA is constitutively released by cancer cells and is found free-floating in patient biofluids (19, 20). Tumor-specific cfDNA increases as tumors grow. The half-life of cfDNA is only 15-120 minutes, making it a valuable and proven biomarker for detecting cancer development in healthy individuals and monitoring tumor response to treatment in cancer patients (21). In 2020, FoundationOne’s Liquid CDx cfDNA test was FDA-approved to detect *EGFR*, *BRCA1/2*, *ALK*, *ATM*, and *PIK3CA* mutations in cfDNA in ovarian, breast, prostate, and lung cancers to stratify patients into treatment regimens (22). Detection of tumor-specific VDJ rearrangements in cfDNA is used to monitor lymphoid lymphoma progression (23–25), and emerging evidence in myeloid malignancies suggests that cfDNA monitoring will be a useful diagnostic tool for leukemia patients as well. For example, in Acute Myelogenous Leukemia, cfDNA assays detected bone marrow relapse 30 days earlier than the standard flow cytometry-based methods (26). Finally, emerging evidence suggests that cfDNA may be more beneficial than cell-based assays in monitoring CNS tumors. For example, in leptomeningeal carcinomatosis, cancer-derived cfDNA was detectable in cases where microscopy did not reveal malignant cells in the CSF, and cfDNA fluctuated over time, correlating with CNS tumor burden (27). A comprehensive examination of several types of CNS malignancies showed that cfDNA isolated from the CSF was much more reliable than genomic DNA isolated from cells within the CSF in identifying tumor-associated mutations (28). These data suggest that cfDNA can identify CNS-associated cancers much more accurately than assays that rely on the presence of cancer cells within the CSF. To our knowledge, cfDNA has not been used to examine MRD status or CNS disease in ALL.

**Figure 1:**
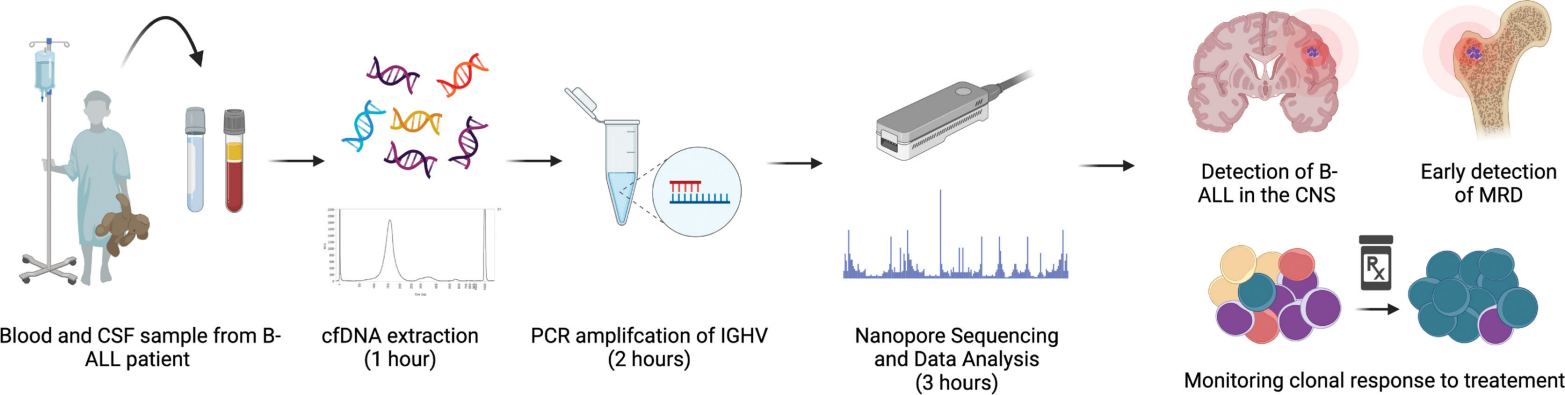
Workflow of IGHV sequencing. Diagrammatical overview of workflow and time input required for sequencing, from patient sample collection to read analysis.

Our study outlines a method for isolation and detection of leukemia-derived cfDNA in the peripheral blood and CNS of pediatric B-ALL patients. We developed a simple and inexpensive workflow based on Nanopore MinION sequencing of PCR amplified VDJ rearrangements in cfDNA, which allowed us to analyze as little as 25 picograms of cfDNA per patient biofluid sample (**Figure 1**). We found that cfDNA can provide a more accurate assessment of B-ALL heterogeneity, as cfDNA sequencing could detect clones not present in the genomic DNA of bone marrow biopsy samples. Plasma cfDNA samples were used to monitor the response of specific clones to treatment and identified B-ALL clones present in MRD. Additionally, our Nanopore cfDNA sequencing workflow could detect B-ALL associated cfDNA in the CSF of patients that had been clinically diagnosed with CNS disease and in some patients diagnosed as CNS-negative. The clearance of CNS-associated clones was confirmed by the loss of cfDNA in the patient’s CSF sample. Our data demonstrate the utility of cfDNA in assessing B-ALL heterogeneity and in monitoring B-ALL progression. The low cost and ease of use make the MinION cfDNA workflow ideal for cfDNA analysis in research settings, and the specificity and sensitivity of the assay in detecting MRD and CNS disease suggest that cfDNA may ultimately be a valuable complement to current cell-based clinical assays.

## Materials and methods

### Isolation and cryopreservation of mononuclear cells from bone marrow aspirate

Bone marrow aspirate was collected in Streck tubes (Streck, Cat # 218962) at the University of Kentucky’s Pediatric Oncology clinic. Samples were diluted with an equal volume of room temperature 2% fetal bovine serum (FBS) in 1X PBS. The sample was slowly added to a 50mL Sepmate tube (Stem Cell Technologies, Cat # 85450) containing 15mL of Ficoll-Paque density gradient medium (Millipore Sigma, Cat # GE17-5446-52) and centrifuged at 2800rpm for 10 min. The top layer containing the mononuclear cells was poured into a new 50mL conical tube. The volume was brought up to 50mL with RPMI supplemented with 10% FBS and spun for 15 mins at 1,400 rpm at 4°C. Media was aspirated, cells were resuspended in 1mL RPMI, then counted with a hemocytometer. An additional 49 mL of complete RPMI was added to the cells, and cells were centrifuged for additional 10 mins at 1,400 rpm at 4°C for the second wash. The media was removed, and the cells were resuspended in 1ml of freezing medium (90% FBS + 10% DMSO) per 10^7^ cells and added to a cryovial. Vials were placed in a CoolCell (Corning, Cat # 432003), cooled at -1°C/minute at -80°C then moved to liquid nitrogen for long-term storage.

### Isolation of genomic DNA from banked mononuclear cells

Vials of banked mononuclear cells from patient bone marrow aspirate were warmed to 37°C and transferred to a sterile 15 mL conical tube. Pre-warmed, 25% fetal bovine serum (FBS) in Iscove’s Modified Dulbecco’s Media (IMDM) was added dropwise to the cells at a rate of approximately 2-3 seconds/ml for 10 mL. Cells were centrifuged at 1,000 rpm for 10 minutes, and the supernatant was gently aspirated. The addition of media, centrifugation, and removal of supernatant was repeated. Cells were then washed once in 1X PBS. gDNA was isolated using the Zymo Quick-gDNA Miniprep Kit (Zymo, Cat # D3025), using the manufacturer’s instructions.

### Isolation of cfDNA from Plasma and Cerebrospinal Fluid

Patient blood and CSF samples were collected into cell-free DNA collection tubes (Streck, Cat # 218962). Samples were stored at room temperature and processed within two weeks of collection. Blood samples were centrifuged at room temperature for 10 minutes at 1,600xg. After the initial spin, the plasma was removed, being careful not to disturb the buffy coat layer, transferred into a clean 1.5 mL Eppendorf tube, and centrifuged for 10 mins at 16,000xg at room temperature. The supernatant containing the cfDNA was carefully removed so as not to disturb the cell pellet and placed in a fresh tube. cfDNA from the plasma was isolated using the Qiagen QIAmp MinElute ccfDNA Midi Kit (Qiagen, Cat # 55284) as per the manufacturer’s instructions, except that the final elution occurs with nuclease-free water pre-warmed at 56°C for an enhanced yield of DNA from the column. cfDNA was isolated from CSF samples using the Zymo Research Quick-cfDNA Serum and Plasma Kit (Zymo, Cat # D4076) according to the manufacturer’s instructions except with the following modifications: i) after adding proteinase K, the samples were incubated at 55°C for 1hr and ii) final elution step was carried out in 35 µl of nuclease-free water pre-warmed at 56°C. cfDNA samples were quantified using a Qubit Fluorometer using the high-sensitivity dsDNA quantification kit (Thermofisher Scientific, Cat # Q32851) according to the manufacturer’s instructions and stored at -20°C.

### PCR amplification of *IGH* regions in genomic and cell-free DNA

The IdentiClone *IGH* Gene Clonality gel detection kit (*In vivo*scribe, 91010020) was used to amplify the immunoglobulin heavy chain (*IGH*) region of genomic and cfDNA. The PCR was performed using the primer master mix labeled Tube A (*In vivo*scribe, Cat # 21010010CE) according to the manufacturer’s directions, with 0.5ng of genomic DNA or cfDNA as input. Tonsil DNA (IVS-0000 polyclonal control, Cat # 40920010, supplied in the kit) was used as a negative control as well as for limit of DNA input detection. Amplitaq Gold Taq Polymerase (ThermoFisher Scientific, Cat # N8080240) was used for PCR amplification at 0.2ul per 50ul reaction, and PCR was run for 40 cycles with the cycling parameters of 95°C for 7 minutes of initial denaturation; 40 cycles of 95°C for 45 sec; 60°C for 45 sec; 72°C for 90 sec and final extension of 72°C for 10min. A portion (5µl) of the PCR reaction was run on agarose gel electrophoresis to confirm the amplification before proceeding with the library preparation.

### Library preparation for MinION sequencing

Following the *IGH* clonality, PCR samples were measured on Qubit, and 50 ng of each sample was used as input for the library prep for the MinION sequencing. The PCR Barcoding Kit (Oxford, SQK-PBK004) was used for the library generation according to the manufacturer’s protocol with the following modifications: i) since an amplicon was used as starting material, the initial fragmentation step of the protocol was omitted, ii) half-reaction volumes were used throughout the library generation procedure to conserve materials. Libraries were loaded onto the MinION Nanopore sequencer (Oxford) and were run until all barcodes had a minimum of 4000 reads, which averaged about 1-2 hours.

### Illumina MiSeq analysis

*IGH* variable region was amplified from plasma cfDNA using the *Invivoscribe* kit as described above. The PCR reaction was size-selected using Ampure bead purification (Beckman Coulter, Cat # A63880) and eluted in nuclease-free water. The eluted DNA was sent to Genewiz for targeted amplicon-based sequencing under their “Amplicon EZ” category. Standard Illumina adapters were used in the library prep, and the final amplicon libraries were run on the Illumina MiSeq sequencing system. The merged Fastq files were then analyzed similarly to the files from the MinION, described below, to assess the *IGH* variable region clonal rearrangements and their abundance.

### MinION analysis pipeline to identify *IGH* rearrangements

Sequencing reads were base-called and demultiplexed using the MinKNOW software package and built-in basecaller. Reads with a quality score of ≥ 7 were used to generate fastq files and for all downstream analyses. Fastq output files were concatenated into a single file per sample. Merged fastq files from MinION and those generated by MiSeq were then processed using the Galaxy server and available tools. Reads were mapped to hg38 using Minimap2 to generate BAM alignment files. Alignment files were then used as input for Feature Counts along with a gff reference file of the *IGH* locus on Chromosome 14. The output text file of how many reads mapped to each feature in the gff reference file was used for data analysis and graph creation. Read counts for each feature were normalized as a percentage of total reads for each patient. Major *IGHV* clones were identified in the diagnosis sample as those features that contained reads equal to or greater than 5% of the total reads for that sample. The select major clones were tracked through subsequent time points in each patient. All graphs were generated using Prism GraphPad

## Results

### Characteristics of B-ALL patients

Pediatric B-cell acute lymphoblastic leukemia (B-ALL) patients were admitted to the UK Healthcare Kentucky Children’s Hospital at initial diagnosis of B-ALL and enrolled for sample collection. We retrospectively chose samples for use in this study based on CNS disease and MRD status after initial therapy. Our cohort consisted of five patients, out of which one patient (AAL-008) was MRD-positive at the end of induction. Patient AAL-004 had a few leukemic blasts noted but was MRD-negative as blast counts did not reach the threshold for a clinical MRD diagnosis. Patients AAL-009, 010, and 012 were MRD-negative at the end of induction, and patients AAL-008 and 010 were positive for CNS disease at the time of diagnosis. **Table 1** summarizes the complete characteristics of these patients. Genomic DNA was isolated from the bone marrow aspirate collected from each B-ALL patient at diagnosis. Cell-free DNA was isolated from plasma and cerebrospinal fluid (CSF) samples collected throughout treatment.

**Table 1:** Patient characteristics.

Subject ID	Blast Count at Diagnosis (*10^9^/L)	WBC Count at Diagnosis (*10^9^/L)	Number of Days to Clear Circulating Blasts	EOI MRD Status	CNS Disease Status	Number of Days to Clear CSF
*ALL-004*	0.57	3.33	9	Negative*	Negative	N/A
*ALL-008*	1.53	4.79	9	Positive	Positive	8
*ALL-009*	14.92	19.38	11	Negative	Negative	N/A
*ALL-010*	1.58	6.32	13	Negative	Positive	9
*ALL-012*	23.88	31.84	11	Negative	Negative	N/A

*Negative MRD at the threshold of one blast per 10,000 cells by flow cytometry, but a few leukemic cells noted.

WBC, white blood cell; EOI, end of induction; MRD, minimal residual disease; CNS, central nervous system; CSF, cerebrospinal fluid.

### Identification of B cell clones *via* Nanopore sequencing of the recombined IGH gene

B cells recombine more than 85 gene segments in the immunoglobulin heavy chain (*IGH*) locus as part of their normal maturation. This locus is also enriched for single nucleotide variants, which further enhances the diversity of the sequence *IGH* region between individual B cells. Sequencing the *IGH* region in genomic DNA has been used to trace B cell lineages and track B-cell malignancies (29–31). We developed a novel experimental workflow that combines PCR amplification of the *IGH* region with the third-generation Oxford Nanopore MinION sequencer (ONT) (32) to detect B-ALL patient-specific *IGH* clonal rearrangements. We focused on analyses of the unique patient-specific variable region rearrangements (*IGHV*) and tracked these clones throughout the treatment. Ultimately, this method can be applied to genomic DNA isolated from blasts in patient samples or cfDNA collected from patient biofluids. In the first step, we used a commercially available PCR kit to amplify the *IGH* sequence between the conserved framework region 1 (FR1) and the conserved Joining (J) region, which allows for a complete representation of all variable regions within the *IGH* gene (referred to hereafter as *IGHV*) (33). PCR products were subjected to library preparation to add adapters and barcodes that allow for multiplexing, and then samples were run through the benchtop MinION Nanopore sequencer. We used a simple pipeline comprised of freely available software to analyze the output files: i) MinKNOW, which operates the sequencer and automatically performs simultaneous sequencing, demultiplexing, and barcode trimming using the built-in Guppy toolkit, ii) Minimap2 to map the merged fastq files to create BAM alignment files, and iii) Featurecounts to determine how many reads align with the *IGHV* region. We have provided step-by-step instructions for analysis using these tools in the Supplemental Methods. From sample collection to data analysis, the entire process takes approximately 6 hours, with most of that time hands-off, costs less than $30 per sample, and can be carried out by users with basic computer skills.

### Limit of input DNA detection on Nanopore workflow

**Figure 2:**
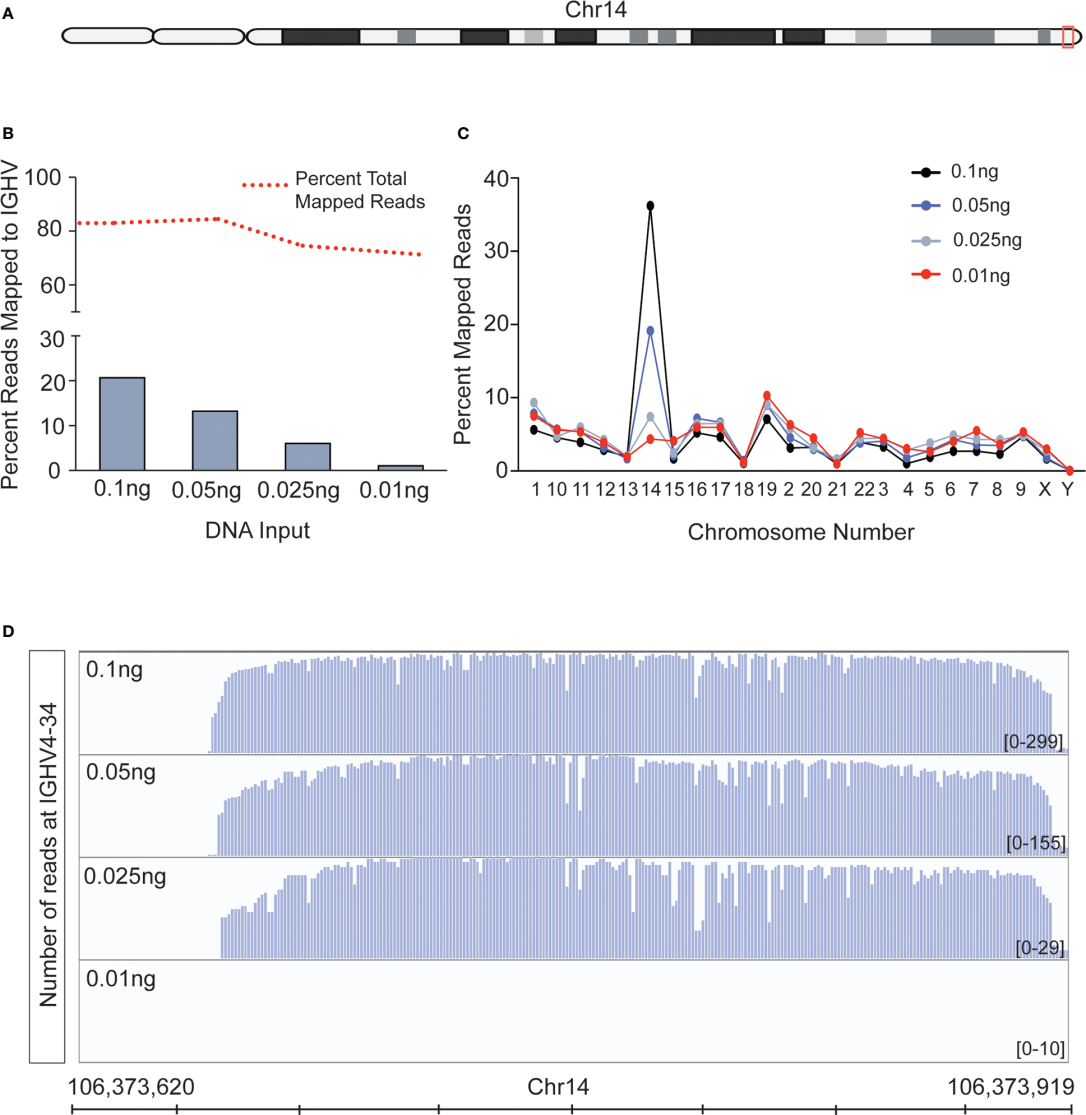
The MinION sequencing workflow can reliably detect IGHV reads in as little as 25 picograms of DNA. **(A)** Schematic of chromosome 14. The red box denotes the region containing the IGHV sequence to which all MinION reads were aligned. **(B)** The IGHV region was PCR amplified from the indicated input amount of normal tonsil DNA. Approximately 4,000 reads were obtained from the resulting libraries using nanopore MinION sequencing. The red dashed line shows the percent of all reads mapped to the human genome, and the bars denote the percentage of those mapped to the IGHV sequence. **(C)** The percent of all reads mapped from the samples in **(B)** are plotted across all chromosomes. **(D)** Alignment plots to a selected IGHV, V4-34, show accurate reads alignment when the input DNA is as little as 0.025ng, or a threshold of 20 reads mapped to IGHV over other location.

The recommended amount of input DNA for *IGH* PCR is 2 ng (34). As our ultimate goal was to adapt our workflow toward the detection of cfDNA, we determined the lowest level of input DNA that could be used in *IGH* PCR and Nanopore sequencing. We tested our workflow on 0.01 - 0.10 ng of genomic DNA isolated from healthy tonsils, which are enriched in B cells. The human *IGH* locus region is located on the long arm of Chromosome 14 (Chr14, **Figure 2A**), and we used the Nanopore generated reads that mapped to this locus over other locations as a readout of PCR specificity and sensitivity. Libraries of *IGH* PCR reactions from all DNA concentrations tested had reads that mapped to chromosome 14. Predictably, mapping frequency to Chr14 decreased with decreasing input DNA amount; however, we did observe reduced mapping in total mapped reads after we lowered DNA input to 0.025ng (**Figure 2B**). When we distributed mapped reads across all chromosomes, it became evident that non-specific mapping increased inversely with template amount. The signal-to-noise was indistinguishable at concentrations below 0.025ng, as reads mapping to Chr14 dropped below the levels of mapping to other chromosomes (**Figure 2C**). Nevertheless, even with the lowest input DNA amount, we could detect reads mapping to the identical *IGHV* clones identified with the highest DNA input amount (**Figure 2D**), suggesting that sensitivity with Nanopore sequencing can be maintained even when specificity decreases. This aspect of Nanopore sequencing is essential when working with patient samples after the treatment regimen to detect MRD when the genetic material obtained may be too low to quantify by standard means.

### cfDNA from patient biofluids as input for Nanopore sequencing workflow

cfDNA is emerging as a robust prognostic biomarker that can assess tumor heterogeneity and tumor burden (35). Additionally, cfDNA is localized to patient biofluids (36), so sample collection is less invasive than tumor or tissue biopsy. cfDNA has not previously been assessed in the B-ALL disease setting. We assessed the Bioanalyzer traces of cfDNA samples isolated from the plasma of patients AAL-004 and AAL-010, and they matched the expected cfDNA sizes of ~150 base pairs (37, 38) (**Supplemental Figure 1**). We applied PCR and Nanopore sequencing of *IGH* to both the genomic DNA (gDNA) isolated from bone marrow aspirate and cfDNA isolated from the plasma at diagnosis. Only sequences that had an 80% or greater match to the *IGH* region of Chr14 were considered B-cell “clones” and included for further analyses. Clones that comprised ≥ 5% of the total mapped reads were considered significant clones in the sample (**Supplemental Table 1**). We reasoned that a ≥ 5% representation of a particular *IGHV* sequence in the sample would most likely be due to clonal expansion of the B-ALL. However, our methods record sequencing data from all clones. If a rare clone emerges as a dominant driver of B-ALL progression at later stages, we can retrospectively trace this clone in banked patient samples.

### Clone distribution is consistent regardless of the sequencing platform

**Figure 3:**
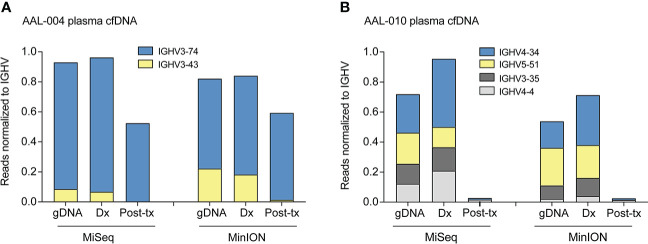
Comparison of clone distribution between Illumina and Nanopore MinION sequencing platforms. IGHV PCR of select time points from patients AAL-004 **(A)** and AAL-010 **(B)** was divided and sequenced with MinION (nanopore) or MiSeq (Illumina). V-region clones comprising more than 5% of the total clones in the sample taken at diagnosis were tracked across the two later time points and normalized to the total number of reads aligned to Chr14 per sample. Clone distribution across different time points within each patient is uniform regardless of the sequencing platform. Dx: sample taken at B-ALL diagnosis, post-tx: sample taken post-treatment.

We used the same cfDNA PCR reaction from the plasma of two patients, AAL-004 and AAL-010, to determine if Nanopore sequencing is comparable to Illumina MiSeq analysis, a routinely used NGS platform for MRD monitoring. Both platforms detected similar clone identity and clonal abundance across treatment time points (**Figure 3**). These data indicate that the Nanopore sequencing workflow can assess the *IGH* clonal repertoire in B-ALL and identify clones in cfDNA, but with a faster turn-around time lower cost than Illumina.

### Nanopore sequencing of *IGH* from cfDNA monitors therapeutic response

**Figure 4:**
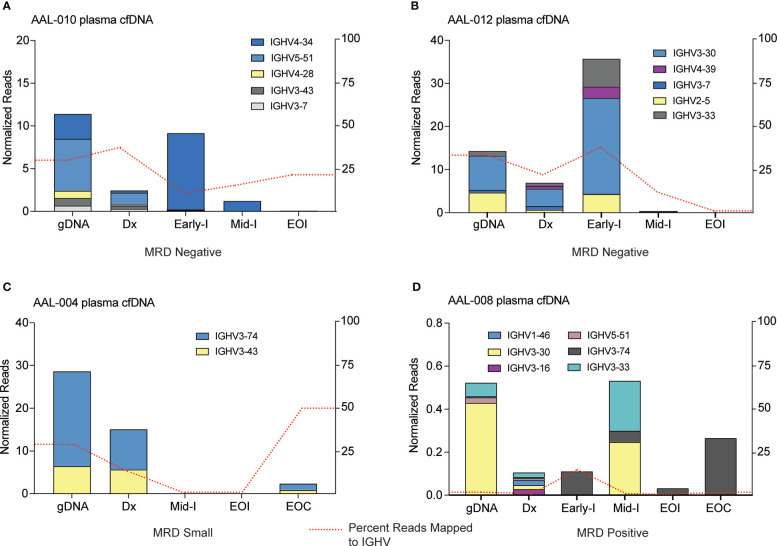
IGHV sequencing of cfDNA can define B-ALL heterogeneity and track clonal response throughout treatment. cfDNA was extracted from plasma samples of four patients, IGHV regions were PCR amplified, and resultant libraries were sequenced using MinION. Data were analyzed using Galaxy. Clones present in the genomic DNA (gDNA) and cell-free DNA plasma samples at diagnosis (Dx) that comprised at least 5% of total reads were tracked across treatment timepoints. Clone abundance is normalized to total mapped reads per library and shown on the left axis. The percentage of reads aligned to the IGHV region on Chr14 (red dashed line) is a relative indicator of leukocyte abundance at each timepoint/sample and is shown on the right axis. Patients in **(A, B)** were clinically diagnosed as MRD negative *via* flow cytometry. Patient **(C)** was MRD negative but had blasts present in the sample that did not reach the clinical cut-off of one blast per 10,000 cell thresholds for MRD diagnosis. Patient **(D)** was diagnosed as MRD positive.

The half-life of cfDNA is 1-2 hours (39–41), making it an excellent biomarker for assessing leukemia burden and analyzing the response of individual clones to therapy. We analyzed the gDNA from bone marrow biopsy and the cfDNA from plasma collected from patients at diagnosis to identify the expanded clones comprising the B-ALL, as described above. These clones were then tracked in subsequent plasma cfDNA samples to assess the changes in clonal distribution throughout treatment. cfDNA samples were taken ~ 1 week into induction chemotherapy (denoted as Early-I in **Figure 4**), ~2 weeks into induction therapy (denoted Mid-I), and at the end of induction chemotherapy (denoted EOI). In some cases, we collected cfDNA at the end of consolidation (EOC) therapy, which can occur several months after diagnosis.

Two patients, AAL-010 and AAL-012, were clinically diagnosed as MRD negative at the end of induction therapy. At their initial disease diagnosis, both patients had heterogenous B-ALL, with 5 distinct *IGHV* sequences present as ≥ 5% of the total mapped reads. Interestingly, in patient AAL-010 we observed a clonal population (*IGHV*4-34) that expanded at the onset of induction therapy (**Figure 4A** **Early-I**) and was eliminated by the end of induction, indicating clonal response to therapy. All identified clones were eliminated after treatment, consistent with this patient’s clinical diagnosis of MRD-free (**Figure 4A**). However, we did detect reads mapping to chr14 within the cfDNA sample taken at the end of induction therapy, (red dashed line, **Figure 4A**), although none of these new clones mapped to any of the ALL clones present at diagnosis. This spike might be attributed to a clonal expansion of a B cell caused by an illness or other antibody response in the patient.

AAL-012 had a clone, *IGHV*4-39, not present in the diagnosis gDNA bone marrow biopsy sample but identified in the diagnosis cfDNA plasma sample (**Figure 4B**). Because every B-ALL clone release cfDNA into the plasma, cfDNA may ultimately be more accurate in detecting the actual clonal composition of the B-ALL than the bone marrow biopsy, which can only detect clones present at the site of biopsy. Additionally, early into both patient AAL-10’s and AAL-012’s treatment, we observed a sharp increase in cfDNA associated with these clones, which likely indicates leukemia cell death and release of DNA into the blood and may indicate a good response to treatment.

Two additional patients, AAL-004 and AAL-008, had blasts present in their clinical samples at the end of induction therapy. Patient AAL-004 was not diagnosed with MRD, as the blast count fell below the one blast per 10,000 cell thresholds for clinical MRD. Patient AAL-008 was MRD positive. In both patients, we detected heterogenous B-ALL comprised of several distinct *IGHV* sequences in the gDNA from bone marrow biopsy and the plasma cfDNA isolated at diagnosis. Patient AAL-004 appeared to clear their clones during induction therapy, as we could not detect reads linked with chr14 in the cfDNA samples. However, we observed clone *IGHV*3-74, a major clone present at diagnosis, re-emerge in cfDNA samples collected at the end of consolidation therapy (**Figure 4C**). Patient AAL-008 unfortunately never cleared the clones detected in the biopsy gDNA and cfDNA sample at diagnosis. Interestingly, we observed that some clones (*IGHV*3-30, *IGHV*3-33, *IGHV*3-74), which were equally distributed amongst the other clones at diagnosis, became enriched at later treatment stages (**Figure 4D**). Although reads from clone *IGHV*3-30 and *IGHV*3-33 drop off during the end of induction (EOI), clone *IGHV*3-74, remains unresponsive. Together, these data highlight the utility of cfDNA analysis as a method to track B-ALL patient-specific clonal dynamics during treatment. This workflow may be useful in identifying patients whose B-ALL does not respond well to therapy, such as patient AAL-008, which may allow them to be stratified into a high-risk category early into their treatment for better disease management. Additionally, cfDNA can be used as a non-invasive method to routinely assess patients for clone re-emergence after treatment *via* simple blood draw.

### cfDNA can be detected in the CSF of B-ALL patients using the MinION sequencing workflow

**Figure 5:**
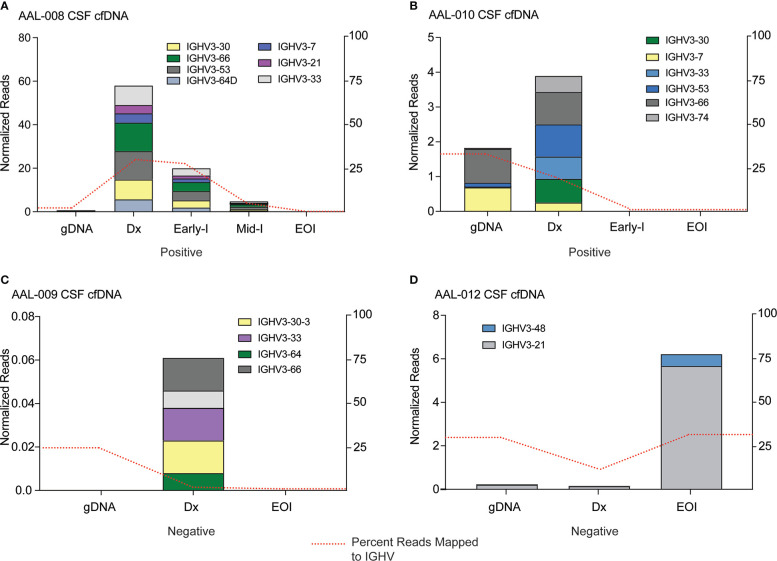
IGHV sequencing can detect cfDNA in the cerebrospinal fluid of B-ALL patients. cfDNA was extracted from CSF samples of four patients, IGHV regions were PCR amplified, and resultant libraries were sequenced using MinION. Data were analyzed using Galaxy. Clones in the genomic DNA (gDNA) of bone marrow biopsy and cell-free DNA (Dx) CSF samples at diagnosis that comprised at least 5% of total reads were tracked across all timepoints. Clone abundance is normalized to total mapped reads per library, shown on the left axis. The percentage of reads aligned to the IGHV region on Chr14 (red dashed line) is a relative indicator of leukocyte abundance at each timepoint/sample and is shown on the right axis. Patients in **(A)** and **(B)** were clinically diagnosed as CNS positive by flow cytometry, indicating infiltration of B-ALL cells into the CNS. Patients **(C)** and **(D)** were diagnosed as CNS negative, although IGHV reads from cfDNA were detectable in the CNS sample.

Approximately 4% of B-ALL patients will have infiltration of ALL blasts into their CNS at diagnosis, and 30-40% of the patients will have pronounced CNS disease at relapse (5). CNS infiltration is a significant adverse prognostic indicator in B-ALL, but the only way to diagnose it is to detect blasts in the CSF sample. However, B-ALL cells can be embedded in the brain or associated with spinal neurons and not be freely floating and detectable in the CSF (5, 42, 43), meaning patients might have CNS disease but present clinically as negative for CNS infiltration. For this reason, every ALL patient is given intrathecal chemotherapy. We reasoned that since cfDNA is released by B-ALL cells into patient biofluids, it might be detectable in CSF samples even if the blasts themselves are not. We applied our MinION sequencing workflow to cfDNA isolated from CSF samples taken from patients diagnosed as CNS positive (AAL-008 and 010) and CNS negative (AAL-009 and 012). We detected *IGHV* reads in the cfDNA from CNS positive B-ALL patients (**Figures 5A, B**), and these clones were cleared by the end of induction therapy. Interestingly, we detected very low levels of *IGHV* sequence in the diagnosis CSF cfDNA sample from patient AAL-009 (**Figure 5C**), even though this patient was diagnosed as CNS negative. Finally, patient AAL-012 was diagnosed as CNS negative. However, we observed an *IGHV* clone *IGHV*3-21 in the cfDNA from the CSF of this patient at diagnosis that significantly expanded by the end of induction (**Figure 5D**). CSF is not drawn from patients after induction therapy when their initial diagnosis is CNS negative, so we could not follow up on the *IGHV* in the cfDNA in later CSF samples of AAL-012. However, their plasma cfDNA remained clear of *IGHV* reads throughout consolidation. Finally, across all patients, we observed that some of the *IGHV* reads in the cfDNA from the plasma were also present in the cfDNA in the CSF, indicating B-ALL clones were present at both locations. In other instances, clones were exclusively present in one location, suggesting that cfDNA analysis may be useful in research focused on ALL homing to the CNS.

## Discussion

Despite advancements in the treatment of Acute Lymphoblastic Leukemia, relapsed leukemia remains the second leading cause of death in pediatric cancer patients (44). The current methods of MRD monitoring in patients’ post-chemotherapy have room for improvement. For example, cell-based quantification *via* flow cytometry has poor resolution. ClonoSeq NGS analysis is costly, can take days to weeks to return results, and is not yet used to detect CNS disease in B-ALL (9, 45, 46). Early detection of MRD, better biomarkers to monitor treatment response in real-time, and a non-invasive, routine screening method to identify ALL recurrence could improve patient disease management. cfDNA analysis has emerged as a robust, minimally invasive prognostic biomarker that correlates well with the tumor heterogeneity and tumor burden. In this study we present a workflow for analyzing cfDNA in the blood and CSF of ALL patients, which, as far as we know, has previously not been applied to B-ALL. Our study demonstrates the utility of MinION-based Nanopore sequencing of *IGH* rearrangement from the cfDNA to quickly visualize B-ALL clonal heterogeneity and track clonal response to treatment from patient biofluids. Towards this end, our workflow is very amenable in terms of small input material for PCR, quick turnaround time, and is very cost-effective. Workflows from other NGS platforms routinely used for cfDNA or MRD detection, such as Illumina, require specialized instruments, batch runs, long wait/turnaround times, and complex pipelines for data analysis. The MinION sequencing device is inexpensive, and the data analysis we describe is an easy-to-use pipeline that utilizes freely available tools such as Nanopipe, Galaxy, and IGV genome browser. These features make the MinION sequencing for the detection of cfDNA a promising platform in the clinical setting.

With a limit of detection as low as 25 picograms of DNA, this workflow is an ideal alternative for cell-based assays requiring genomic DNA from clinical samples limited in material **(** **Figure 2** **)**. Our workflow identified similar clonal heterogeneity and abundance compared to routinely used and more expensive NGS platforms such as Illumina Miseq, indicating the robustness of our assay **(** **Figure 3** **)**.

Nanopore sequencing revealed interesting B-ALL clonal dynamics from our patient cohort that had a clinical diagnosis of either MRD positive (ALL-008) or negative (AAL-010 and AAL-012) and MRD small (AAL-004). All the patients showed heterogenous B-ALL in that several distinct *IGHV* sequences were present. We were able to track the response of these individual clones throughout the treatment and identified clones that were either responded to or survived chemotherapy. In a research setting, these data could be combined with other NGS methods, such as single-cell RNAseq, and may provide new insights into mechanisms of chemotherapy resistance. Interestingly, analysis of cfDNA from plasma samples at patient diagnosis identified *IGHV* sequences and B-ALL clones that were not present in the cell-based analysis of the genomic DNA collected from the bone marrow biopsy sample **(** **Figure 4** **)**. These data reiterate the ability of cfDNA to act as a biomarker that can capture the full extent of heterogeneity and clonal dynamics within a patient’s leukemia. Although, we were able to detect multiple *IGHV* clones in each of the B-ALL patients, we realize our analysis may not completely capture the extent of *IGH*V-D-J rearrangements. Since the hg38 reference genome does not include *IGH* recombinations, mapping of the shorter, *IGH* joining (J) or diversity (D), regions will be less accurate because they exist up hundreds of kilobases away from the variable regions (V). Thus, we focused on the longer *IGHV* regions since they would be the most likely to capture clonal heterogeneity. For a more quantitative analysis of possible rearrangements of *IGHV* clones with different J and D regions the raw read data from MinION can be used in other freely available VDJ analysis software such as IMGT/V-Quest (47, 48) or VDJServer (49).

Because ALLs have a low mutational burden, cell-based clonal analyses like ClonoSEQ are the only NGS methods available for sensitive detection of MRD. ClonoSEQ is currently completed only at the end of the induction chemotherapy. Longitudinal Nanopore analysis of cfDNA with every blood draw could provide a valuable complement to ClonoSEQ, allowing for a more complete assessment of ALL responses throughout a patient’s treatment. For example, a caveat of our assay and ClonoSEQ is that it cannot distinguish between B-ALL and normal B cell expansion due to an antibody response, such as during an infection. We detected reads mapping to chr 14 in patient AAL-010 at EOI **(** **Figure 4A** **)**. None of these reads mapped to any clones identified at diagnosis, suggesting an antibody response that presents during transient infection rather than B-ALL recurrence. Since we can run our workflow on the next blood sample from the patient, we can easily confirm whether this expansion was indeed an immune response or a cause for clinical concern.

Another important use of our workflow is the detection of CNS positive disease in B-ALL. Blasts can spread into the CNS during B-ALL development, and the CNS is a common site for ALL relapse (50). Outcomes in B-ALL patients with CNS positive disease are lower than those without evidence of CNS positivity. Patients typically have 16-20 lumbar punctures (LP) with administration of intrathecal chemotherapy. With each lumbar puncture (LP) CSF is assayed by cell count and cytospin to surveille for evidence of CNS relapse. Cytospin analysis requires a pathologist review a concentrated CSF prep under microscopy, manually enumerating the quantity of each cell type, which is an arduous process. A primary clinical concern is that current diagnostic methodologies may not be sensitive enough to detect CNS disease in ALL, as blasts may be embedded with CNS tissue and not present in CSF samples (43, 51). Interestingly, we detected cfDNA in the CSF of one patient diagnosed as CNS negative, meaning cells were not detected by flow cytometry of the CSF sample. This finding attests to the potential sensitivity of our cfDNA workflow in monitoring CNS disease. Two of our patients (AAL-008 and AAL-010) were diagnosed with CNS disease. Nanopore sequencing allowed us to identify which clones spread into the CNS versus those that remained in the blood. The absence of further cfDNA reads in CSF samples after the initial doses of intrathecal chemotherapy suggested that those clones were cleared from the CNS **(** **Figure 5** **)**. CSF is removed from patients each time they receive intrathecal chemotherapy to prevent increased CSF volume when the treatment is injected. Instead of discarding this fluid, cfDNA analysis could provide valuable insights into CNS disease status and clonal response.

Although our conclusions are based on a very small sample study, the observed data on *IGHV* sequencing of cfDNA and MRD status suggests a possible correlation. Similarly, *IGHV* sequencing showed obvious contrasts between CNS-positive and CNS-negative patients. A future study with 20 to 30 patients will be sufficient for proof of concept to show that cfDNA assays may be useful in detecting the presence of ALL in patients when blasts are not in the biofluid sample.

In summary, our study demonstrates the utility of a Nanopore sequencing workflow as a simple, rapid, and low-cost method to visualize B-ALL patient-specific *IGHV* clonal heterogeneity and track these clones throughout treatment. We found that quantitation of *IGHV* reads from cfDNA samples provided insights into B-ALL heterogeneity in our patient cohort. The workflow could be scaled up for quickly and efficiently assessing the dynamics of B-ALL clones in response to treatment in large patient datasets, particularly in Consortia studying B-ALL. Similar Nanopore sequencing strategies could also be developed and applied to detect tumor-associated mutations in cfDNA in any cancer type.

## Data availability statement

The raw read data from the MinION sequencing are uploaded to the Sequence Read Archive (NCBI) Project ID:PRJNA891254. Further inquiries can be directed to the corresponding author.

## Ethics statement

The studies involving human participants were reviewed and approved by protocol 44672, approved by the University of Kentucky’s Institutional Review Board. Written informed consent to participate in this study was provided by the participants’ legal guardian/next of kin.
