# Supplementary material for: Nanopore sequencing of clonal IGH rearrangements in cell-free DNA as a biomarker for acute lymphoblastic leukemia
Source: Front Oncol. 2022 Dec 14;12:958673. doi: 10.3389/fonc.2022.958673 (PMC9795051; doi:10.3389/fonc.2022.958673)
Supplement: Supplementary file 1 [file DataSheet_1.pdf]

Supplemental Figures

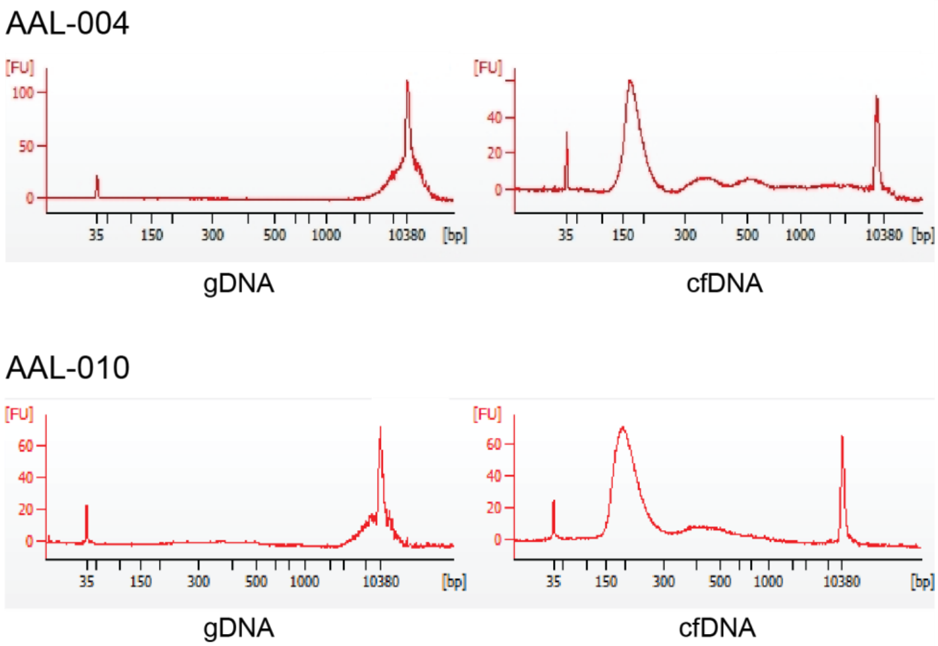

Supplemental Figure 1. Electropherograms distinguish genomic DNA from cfDNA. Genomic DNA (gDNA) and cell-free DNA(cfDNA) samples purified from the diagnosis bone marrow biopsy and blood draw from patients AAL-004 and AAL-010 were run in an Agilent Bioanalyzer 2100 using a high-sensitivity DNA kit. Genomic DNA appears as sizes >10,000bp (denoted on the X axis), while cfDNA appears as ~150bp peak. The y-axis denotes signal intensity.

Supplemental Methods

Extended Methods for Nanopore Sequence Analysis

Software Packages Required:

1. Account with Galaxy (usegalaxy.org)

2. Minimap2 (hosted by Galaxy)

3. FeatureCounts (hosted by Galaxy)

Input Files Required:

1. .fastq read files from a sequencing experiment. These should be one file per experiment. If experiment generates multiple files, merge them into one before proceeding.

2. hg38.IGHV.gff3 feature file. This file annotates the different genomic features in the IGHV region (i.e., genes, exons, etc) and their corresponding genomic positions (i.e., Chr14:101,224-115-032). It was created by trimming the Chr14.gff3 file down to the IGHV region.

Analysis Protocol:

A. Minimap2

This software package is used for mapping sequencing data to a reference genome to determine read locations and sequencing accuracy. This particular mapper is optimized for handling long reads, but can just as easily align short-read data.

1. **Log into Galaxy and use the "Upload Data" icon to upload the two input files onto the Galaxy server.** A new window will pop up where you can select the files of interest from a local computer or drag-and-drop them in. Press "start" to begin upload. Correctly uploaded files will appear as green tabs on the left-hand side of the screen.

2. **Search for Minimap2 software by typing it into the search bar in the top right corner. Select the "map with minimpa2" option from the results**. The main window will now show a

graphical user interface for the minimap2 software package which will already have most fields populated with default presets. (Only change presents outlined below.)

3. **Select a Reference Genome**. In the drop-down menu of "Using a reference genome" select "Human (Homo sapiens) (b38):hg38". This is the most recent version of the human reference genome that you will use to map your reads.

4. **Select input file for mapping**. In the "Select fastq dataset" drop-down menu tab pick your .fastq input file. (If the file does not show up in the drop-down menu click the open file folder icon to the right of it to select it manually).

5. **Select Mapping Parameters**. In the "Select a profile preset options" drop-down menu pick the third option of Oxford Nanopore read to reference mapping.

6. **Select output format**. Expand the "Set advanced output options" tab and double check that the output format is set to BAM (not CRAM or PAF).

7. **Press Execute to run job**. The job will appear in the right-hand column as grey while it's in the queue and will turn pink when it begins processing. Once it is done it should turn green. This BAM output job will have two files associated with it: a .bam alignment file and a .bai index file. It will also serve as the input for the next step.

B: FeatureCounts:

This software tool counts the number of reads that are aligned at a specific annotated feature. It requires a read alignment file (BAM) and a feature reference file (gff or gff3) that it cross references against one another.

1. **Search for FeatureCounts software** by typing it into the search bar in the top right corner and clicking on the name in the results.

2. **Select your alignment file.** In the “Alignment file” drop-down menu pick the Minimap2 alignment file from above. (If the file does not show up in the drop-down menu click the open file folder icon to the right of it to select it manually).

3. **Select the Gene Annotation file**. In the “Gene Annotation File” drop-down menu pick the “in your history” option and then select the hg38.IGHV.gff3 file you uploaded earlier in the space that appears.

4. **Specify counting parameters**. Expand the “Advanced options” tab. Here you will specify which features you want to count since a gff file will contain many feature types. In the “GFF feature type filter” put “gene” and in “GFF gene identifier” put “Name”. Case is IMPORTANT here since software searches for exact matches. Scroll down to “Long Reads” option and turn it to Yes.

5. **Press Execute to run job**. Job will appear in the right-hand column. The output will generate two files. One will be the actual counts at every feature selected from the gff file and the second will be a Summary file of read statistics.
